# An Exploratory Study of the Relative Effects of Various Protective Factors on Depressive Symptoms Among Older People

**DOI:** 10.3389/fpubh.2020.579304

**Published:** 2020-11-12

**Authors:** Caitlin Worrall, Michelle I. Jongenelis, Peter M. McEvoy, Ben Jackson, Robert U. Newton, Simone Pettigrew

**Affiliations:** ^1^School of Psychology, Curtin University, Bentley, WA, Australia; ^2^Melbourne Centre for Behaviour Change, Melbourne School of Psychological Sciences, The University of Melbourne, Melbourne, VIC, Australia; ^3^Centre for Clinical Interventions, Perth, WA, Australia; ^4^School of Human Sciences (Exercise and Sports Science), University of Western Australia, Crawley, WA, Australia; ^5^Exercise Medicine Research Institute, Edith Cowan University, Joondalup, WA, Australia; ^6^The George Institute for Global Health: Australia, Newtown, NSW, Australia

**Keywords:** older adults, aging–old age–seniors, protective factors, comprehensive model, depressive symptoms

## Abstract

**Objective:** The present study investigated the relative importance of various factors found to be negatively associated with depressive symptoms in older adults and assessed the potential moderating effect of sociodemographic characteristics for each factor.

**Method:** Depressive symptoms were measured with the Center of Epidemiological Studies Depression Scale. Psychological, social, and physical health measures relating to the following factors were also administered: personal growth, purpose in life, self-esteem, self-efficacy, social support, self-rated health, life satisfaction, and physical activity. Multivariate linear regression analysis was used to investigate the most important factors associated with depressive symptoms, and moderation analyses were employed to identify any moderating effects of sociodemographic factors.

**Results:** Life satisfaction, self-esteem, and purpose in life were found to be negatively associated with depressive symptoms. Only one moderating effect was observed—the negative relationship between life satisfaction and depressive symptoms was significantly stronger among the younger respondents.

**Conclusion:** These findings suggest that strategies for the prevention or amelioration of depressive symptoms across subgroups of the senior population could be optimized by focusing on enhancing life satisfaction, self-esteem, and purpose in life.

## Introduction

Depression is a leading cause of burden of disease worldwide (1). With the proportion of people aged 60+ years worldwide projected to increase from 13% in 2017 to ~21% in 2050 (2), the prevention and amelioration of depressive symptoms among older adults is recognized as a public health priority to ensure increasing life expectancy is accompanied by positive psychological well-being (3). Depressive symptoms can be especially debilitating for older adults because they (i) are particularly intransigent among members of this population segment (4); (ii) complicate the treatment of chronic diseases (5), which are disproportionately prevalent among older adults (6); and (iii) often go undetected due to uncertainty about what constitutes depressive symptoms in this cohort (7, 8). In addition, older adults tend to have more limited social networks and suboptimal coping strategies compared to younger cohorts, which can make them more vulnerable to depressive symptoms (9–11). The development of effective strategies to prevent and ameliorate depressive symptoms is critical to optimize older people's well-being and reduce health system costs (5, 12).

Identifying factors that can protect against later life depressive symptoms is important for informing the development of appropriate prevention and amelioration strategies. Previous research suggests that relevant modifiable factors that could be the focus of such strategies include social support, self-rated health, and physical activity (13–16). There is strong evidence for the protective effects of each of these factors, individually and in various combinations, across both cross-sectional and longitudinal studies (17–26). Overall, research investigating the trajectory of depressive symptoms in older people has found higher levels of social support, self-rated health, and physical activity to be associated with (i) fewer depressive symptoms at baseline and (ii) a lower likelihood of depressive symptoms emerging over time (24, 25). In addition to these well-established protective factors, there is growing evidence that life satisfaction, purpose in life, personal growth, self-efficacy, and self-esteem are protective against depressive symptoms (15, 26–34).

While previous work supports the importance of each of these protective factors, to date there does not appear to be research incorporating them all to provide an understanding of their relative effects to enable appropriate prioritization in intervention design. To address this deficit, the present study adopted an exploratory approach to investigate the relative importance of modifiable factors that have been found to be protective against depressive symptoms (as outlined above). A second aim was to investigate whether sociodemographic characteristics (gender, age, living arrangement, and educational attainment) moderate the relationships between these protective factors and depressive symptoms to assist in identifying specific sub-segments of older adults who are likely to benefit most from interventions. The results provide insights into which factors are likely to be important to consider in the development of population-level depressive symptom prevention and amelioration strategies for older people, and also suggest key variables to incorporate in future longitudinal research designed to further extend this field of research.

## Method

### Design, Recruitment, and Procedure

The data used in this cross-sectional study were collected between 2014 and 2016 as part of a larger project exploring healthy aging among older adults (35). Ethical approval was received from Curtin University's Human Research Ethics Committee. Eligibility criteria were being aged 60 years or older, living in a community setting, and being fully retired. Participants were recruited via a range of methods including notices in community newspapers, radio announcements, and flyers placed at seniors' events and retirement villages across the metropolitan area of Perth, Western Australia.

In total, 801 adults met the above eligibility criteria and provided written informed consent. Participation involved completing a self-administered survey that included psychological, social, and physical health measures validated for use in older adults. These measures are described in detail below. Unless otherwise stated, the items forming each of the scales included in the survey were summed for analysis purposes.

### Sample Characteristics

The final sample consisted of older adults ranging in age from 60 to 95 years (M = 71.93 years, SD = 6.68), 61% of whom were female. Characteristics of the present sample alongside those of the Australian older adult population are presented in Table 1. Pearson chi-square tests indicated the sample was representative in terms of gender, age, and education but not living arrangement: the present sample had a significantly higher proportion of older adults living alone compared to the Australian older adult population.

**Table 1:** Sample characteristics.

**Characteristics (%)**	**Sample (*N* = 801)**	**Australian 60+ population^a^** **(*N* = 4,976,160)**	***P***
Gender			0.317
Female	61	53	
Male	39	47	
Age			0.085
60–69	41	50	
70–79	46	31	
80+	13	19	
Education			0.120
Non-tertiary	43	55	
Tertiary	57	45	
Living arrangement			0.040
Not living alone	65	79	
Living alone	35	21	

*Education = highest level of education attained*.

a *Percentages based on data for all women and men aged 60 years and older from the 2016 Australian Census (36)*.

### Outcome Variable

Depressive symptoms were assessed using the 20-item Center for Epidemiological Studies Depression Scale [CES-D: (37)]. Participants responded to each item (e.g., “I felt that everything I did was an effort”) on a 4-point scale that ranged from 0 (*rarely or none of the time*) to 3 (*most or all of the time*). Cronbach's alpha in the present study was 0.87, indicating good reliability.

### Independent Variables

The 14-item Personal Growth and Purpose in Life subscales of Ryff's (38) Psychological Well-Being Scale were used to measure personal growth and purpose in life, respectively. Responses to items (e.g., “I have the sense that I have developed a lot as a person over time”; “I have a sense of direction and purpose in life”) were made on a scale of 1 (*strongly disagree*) to 5 (*strongly agree*). Cronbach's alpha in the present study indicated good reliability for scores on both the Personal Growth (α = 0.86) and Purpose in Life (α = 0.88) subscales.

Self-esteem was measured using the 10-item Rosenberg Self-Esteem Scale (39). Respondents answered each item (e.g., “I feel that I'm a person of worth, at least on an equal plane with others”) on a 4-point scale that ranged from 0 (*strongly disagree*) to 3 (*strongly agree*). Cronbach's alpha for scores on this scale was 0.88, indicating good reliability. The 24-item Social Provision Scale (40) was used to assess social support. Each item (e.g., “There is someone I could talk to about important decisions in my life”) was measured on a 4-point scale that ranged from 1 (*strongly disagree*) to 4 (*strongly agree*). The scores on this scale were found to have excellent reliability (α = 0.92).

Self-efficacy was assessed using the 10-item General Self-Efficacy Scale (41). Participants responded to each item (e.g., “I can solve most problems if I invest the necessary effort”) on a 4-point scale that ranged from 1 (*not at all true*) to 4 (*exactly true*). Cronbach's alpha was 0.90, indicating excellent reliability. Life satisfaction was assessed by asking participants to rate how satisfied they are with their life on a scale of 1 (*very satisfied*) to 5 (*very dissatisfied*) [adapted from the World Values Survey: (42)]. For analysis purposes, this variable was reverse scored.

Level of physical activity was measured by asking participants: *How many hours of moderate to vigorous activity (that is, physical activity that makes you breathe harder or puff and pant) would you do in an average week?* The definition provided for moderate to vigorous activity was based on the Australian Department of Health's Physical Activity and Sedentary Behavior Guidelines (43). Response options were: 0 h, <1 h, between 1–2 h, between 2–3 h, between 3–4 h, between 4–5 h, and 5 or more h.

Consistent with previous research (44), self-rated health was assessed by asking participants to describe their physical health on a scale from 1 (*very good*) to 5 (*very bad*). For analysis purposes, this variable was reverse scored.

Sociodemographic variables included age (treated as continuous), gender (1 = male, 2 = female; treated as dichotomous), living arrangement (1 = lives alone, 2 = does not live alone; treated as dichotomous), and highest level of education attained (no formal school/primary school, high school, technical/trade certificate, undergraduate, postgraduate; treated as continuous).

### Statistical Analysis

Descriptive statistics for each modifiable protective factor are presented in Table 2. Univariate linear regression analyses were conducted to assess the relationships between each of the independent variables and the outcome variable of depressive symptoms (treated as continuous). Independent variables found to be significantly associated with depressive symptoms were then simultaneously entered into a linear multivariate regression model using SPSS 26. The Variance Inflation Factor (VIF) was used to assess for multicollinearity.

**Table 2:** Descriptive statistics for tested protective factors (IVs) and depressive symptoms.

**IVs**	**M** **(SD)**	**Sample range**	**Scale range**	**Skewness**	**Kurtosis**
Life satisfaction	4.07 (0.82)	1–5	1–5	−0.99	1.19
Self-esteem	23.48 (4.96)	1–30	0–30	−0.68	0.43
Purpose in life	66.57 (11.72)	18–84	14–84	−0.76	0.43
Social support	79.00 (10.41)	33–96	24–96	−0.58	0.49
Self-rated health	4.00 (0.77)	1–5	1–5	−0.99	1.20
Self-efficacy	32.19 (4.38)	12–40	10–40	−0.37	0.77
Physical activity	3.77 (1.82)	1–7	1–7	0.39	−0.94
Personal growth	69.48 (10.06)	29–84	14–84	−0.73	0.27
Depressive symptoms	9.02 (8.14)	0–54	0–60	1.68	3.59

*IVs = independent variables; M = mean; SD = standard deviation*.

Moderation analyses were conducted using the PROCESS macro in SPSS to determine if the sociodemographic variables of gender, age, level of education, or living alone moderated the relationship between each of the significant independent variables and the outcome variable of depressive symptoms (45). Each of the independent variables found to be significant in univariate analyses and each of the sociodemographic variables were entered in analyses separately. Bootstrapping was performed (*n* = 5,000 samples), and a Bonferroni-adjusted alpha level of <0.005 was used to control for the family-wise error rate. Missing data were treated listwise.

## Results

### Regression Analyses

Univariate regression analyses showed that life satisfaction, purpose in life, personal growth, self-esteem, social support, self-efficacy, physical activity, self-rated health, educational attainment, and living arrangement were all negatively associated with depressive symptoms (see Supplementary Table 1 for results of univariate regressions). A multivariate regression analysis combining these variables into a single model was used to explore the relative importance of these factors. VIF was <10 indicating that multicollinearity was not an issue. The model explained 55.6% of the variance in depressive symptoms (*F*_(10, 734)_ = 91.93, *p* < 0.001). The variables in the model that remained significantly and negatively associated with depressive symptoms at the Bonferroni-adjusted alpha level of <0.005 in descending order of effect were: life satisfaction, self-esteem, and purpose in life (see Table 3).

**Table 3:** Unstandardized parameter estimates, standardized parameter estimates, and standard errors for the multivariate model (in descending order of part *r*^2^).

**IVs**	**B**	***SE***	**β**	***p***	**95% CI for B**	**Part *r*^**2**^**
**Life satisfaction**	–**2.76**	**0.33**	–**0.28**	**<0.001**	–**3.40**, –**2.12**	–**0.21**
**Self-esteem**	–**0.42**	**0.06**	–**0.26**	**<0.001**	–**0.54**, –**0.30**	–**0.17**
**Purpose in life**	–**0.13**	**0.03**	–**0.19**	**<0.001**	–**0.19**, –**0.07**	–**0.11**
Social support	−0.07	0.03	−0.08	0.012	−0.12, −0.02	−0.06
Self-rated health	−0.77	0.30	−0.07	0.011	−1.37, −0.18	−0.06
Self-efficacy	−0.13	0.06	−0.07	0.018	−0.25, −0.02	−0.06
Living alone	−0.82	0.45	−0.05	0.066	−1.70, 0.06	−0.05
Physical activity	0.11	0.12	0.03	0.338	−0.12, 0.35	0.02
Personal growth	0.01	0.03	0.01	0.758	−0.05, 0.06	0.01
Education	−0.00	0.19	0.00	0.997	−0.38, 0.38	0.00

*IVs = independent variables; B = unstandardized estimates; SE = standard error of B; β = standardized estimate; p = significance value; CI = confidence interval; Part r^2^ = proportion of unique variance accounted for. Results significant at p <0.005 are presented in bold*.

### Moderating Effects

At the Bonferroni-adjusted alpha level of <0.005, a significant moderating effect of age was observed for life satisfaction (B = 0.13, SE = 0.04, *p* < 0.002, 95% CI for B [0.05, 0.21]). See Supplementary Table 2 for results of moderation analyses. *Post-hoc* investigation of this effect showed that life satisfaction was negatively associated with depressive symptoms for all age groups, but the strength of the association was stronger for those participants below the average age of this sample compared to those of mean age or older (see Table 4 and Supplementary Figure 1 for significant results). Age did not moderate the relationships between any of the other independent variables and depressive symptoms, nor were there significant moderating effects observed for gender, living arrangement, or educational attainment (see Supplementary Table 2).

**Table 4:** Significant moderating effect of age between life satisfaction and depressive symptoms.

**Age**	**B**	***SE***	***p***	**95% CI for B**
65.25 (−1SD)	−6.82	0.35	<0.001	−5.98, −4.35
71.78 (Mean)	−5.99	0.28	<0.001	−6.54, −5.45
78.30 (+1SD)	−5.16	0.41	<0.001	−5.98, −4.35

*B = unstandardized estimates; SE = standard error of B; p = significance value; CI for B = confidence interval. Bonferroni-adjusted alpha level of <0.005*.

## Discussion

To better understand the nature of the relationships between factors that are particularly important in protecting against depressive symptoms among older adults, the present study combined a broad range of factors that have been identified in previous research as being potentially relevant. To assess whether interventions should be targeted at specific subgroups of the wider older adult population, a second aim of this study was to investigate whether sociodemographic characteristics moderated any of the relationships between each protective factor and depressive symptoms.

The tested model explained a large proportion (55.6%) of the variance in depressive symptoms. Life satisfaction, self-esteem, and purpose in life were found to have the strongest (negative) association with depressive symptoms, which is consistent with previous longitudinal research (26, 30–33). However, this past work examined each of these variables in isolation or in models including a limited number of factors. The results of the present study indicate that even when considering a larger number of potential variables, life satisfaction, self-esteem, and purpose in life may be especially important in preventing and ameliorating depressive symptoms among older people.

The results of the present study in relation to physical activity are in contrast with the conclusions of a recent systematic review that found a negative relationship between extent of participation in physical activity and the experience of depressive symptoms among older people (16). The non-significant relationship found here may reflect the range of other psychological variables included in the study and their relative importance in protecting against depressive symptoms. Measurement limitations may also have played a role, such as the reliance on self-report (46, 47) and the assessment of only moderate to vigorous activity, which may not adequately capture all forms of protective activity relevant to older people (18, 21–23).

In terms of the moderation analyses used to assess whether interventions should target particular groups of older adults, just one moderating effect (age) was found, whereby a stronger relationship was found between life satisfaction and depressive symptoms among those participants below the average age of this sample, thus suggesting that interventions to improve life satisfaction could be particularly beneficial for those who are in this younger category (and likely to be newly retired). There is little prior work with which to compare these moderation outcomes. Some studies have examined the moderating effects of age, gender, education level, and living arrangement on the relationship between social support and depressive symptoms (48–51). This work has produced inconsistent results, which in combination with the general lack of effects found in the present study suggests that there may be little need to tailor intervention efforts to demographic subgroups within the broader cohort of older people.

### Implications

The results from this study highlight the importance of life satisfaction, self-esteem, and purpose in life as focus areas for interventions aimed at preventing and ameliorating depressive symptoms among older people. While self-esteem exhibits trait-like stability (52), and is thus better suited to individual therapeutic intervention, life satisfaction and purpose in life have the potential to be modifiable through population-level interventions. As such, interventions that focus on ways of enhancing life satisfaction and purpose in life are likely to hold most potential for scalable prevention and amelioration strategies. Previous research suggests that encouraging people to participate in meaningful tasks such as hobbies, leisure activities, and/or volunteering can increase their life satisfaction and purpose in life (53–56). These activities have been suggested to provide older adults with opportunities that promote purpose in life and life satisfaction from (i) the relationships formed, (ii) the pursuit of goals, (iii) maintenance of independence, and (iv) engagement with the community (56, 57). These types of activities have also been found to be associated with social support, self-efficacy, and self-rated health (58–60). Further, participation in meaningful tasks appears to be beneficial in helping individuals adjust to age-related losses such as retirement (i.e., loss of work role) and bereavement (53, 55, 60). Encouraging older adults to engage in meaningful activities and facilitating relevant opportunities for them to do so may thus constitute means of preventing and ameliorating depressive symptoms in later life.

### Limitations, Future Directions, and Strengths

The main limitation of the present study was its cross-sectional design. Further research is needed to test the results longitudinally to assess whether the identified relationships hold over time. Another potential limitation was the use of convenience sampling, although the resulting sample was largely similar in profile to the population of older Australians, with the exception of living arrangement (see Table 1). Future studies should seek to access representative samples to test whether these results are generalizable. Similar research could also be conducted in other countries to assess the extent to which the identified relationships are relevant to other cultures. Given the inclusion of a broad range of psychological constructs in this study, a further potential limitation was the risk of social desirability bias in responses. To minimize this risk, an “arms-length” data collection method was used in the form of self-administered surveys. This approach has been found to result in more truthful responses to sensitive questions compared to when an interviewer is present (61).

The primary strength of this study was the large number of potential protective factors incorporated into a model that was tested on a substantial sample of community-dwelling older people. However, despite the wide range of factors included in this study, some potentially relevant variables were not assessed and could be incorporated into future research. In particular, including a measure of objective health could provide additional important data and overcome the limitations associated with relying solely on self-rated health as an indicator of physical well-being. Further, recent research suggests that sleep and diet quality may influence older people's experience of depressive symptoms (16), making these potentially important variables to include in future studies.

## Conclusion

This study assessed the relative importance of a large number of factors that have been established in prior work as being protective against depressive symptoms in older adults. Life satisfaction and purpose in life were found to be the most influential factors, and could therefore be the focus of prevention and amelioration strategies targeting depressive symptoms in later life. Previous research has shown that engaging in activities perceived to be meaningful can increase older adults' life satisfaction and purpose in life. As such, it is likely that programs that focus on enhancing these protective factors could decrease the risk of depressive symptoms and improve overall well-being among older adults.

## Data Availability Statement

The datasets presented in this article are not readily available because ethics clearance was received on the basis that information would be confidential. Requests to access the datasets should be directed to spettigrew@georgeinstitute.org.au.

## Ethics Statement

The studies involving human participants were reviewed and approved by Curtin University Human Research Ethics Committee. The patients/participants provided their written informed consent to participate in this study.

## Author Contributions

CW conducted the analyses and took primary responsibility for preparing the manuscript. MJ, SP, and CW collected the data. SP and MJ assisted with manuscript preparation. All authors contributed to study conceptualization, read and edited drafts of the manuscript, and approved the final manuscript. All authors contributed to the article and approved the submitted version.

## Conflict of Interest

The authors declare that the research was conducted in the absence of any commercial or financial relationships that could be construed as a potential conflict of interest.
