# Supplementary material for: An Exploratory Study of the Relative Effects of Various Protective Factors on Depressive Symptoms Among Older People
Source: Front Public Health. 2020 Nov 12;8:579304. doi: 10.3389/fpubh.2020.579304 (PMC7690559; doi:10.3389/fpubh.2020.579304)
Supplement: Supplementary file 1 [file Data_Sheet_1.docx]

**Supplementary Material**

Table S1.

*Univariate Regression Analyses between Independent Variables (IVs) and Depressive Symptoms*

| IVs | B | *SE* | β | *p* | 95% CI for B |
| --- | --- | --- | --- | --- | --- |
| Life-satisfaction | -6.11 | 0.28 | -0.62 | .000 | -6.66, -5.58 |
| Self-esteem | -1.04 | 0.05 | -0.64 | .000 | -1.13, -0.95 |
| Purpose in life | -0.44 | 0.02 | -0.62 | .000 | -0.47, -0.40 |
| Social support | -0.40 | 0.02 | -0.52 | .000 | -0.452, -0.36 |
| Self-rated health | -4.04 | 0.35 | -0.38 | .000 | -4.73, -3.35 |
| Self-efficacy | -0.84 | 0.06 | -0.45 | .000 | -0.96, -0.72 |
| Personal growth | -0.34 | 0.03 | -0.42 | .000 | -0.40, -0.29 |
| Physical activity | -0.63 | 0.16 | -0.14 | .000 | -0.95, -0.32 |
| Living alone | -2.44 | 0.61 | -0.14 | .000 | -3.63, -1.25 |
| Education | -0.96 | 0.27 | -0.13 | .000 | -1.49, -0.43 |
| Gender | 1.09 | 0.60 | 0.07 | .068 | -0.08, 2.26 |
| Age | -0.04 | 0.05 | -0.03 | .354 | -0.13, 0.05 |

*Note.* IVs = independent variables; B = unstandardized estimates; SE = standard error of B; β = standardized estimate; p = significance value; CI = confidence interval. Results significant at p < .05 included in multivariate analyses.

Table S2.

*Moderating Interactions between the Significant Independent Variables (IVs) and Depressive Symptoms*

|  | Interactions | | | | | | | | | | | |
| --- | --- | --- | --- | --- | --- | --- | --- | --- | --- | --- | --- | --- |
| Moderators | Age | | | Gender  (1 = male, 2 = female) | | | Living Arrangement  (1 = lives alone, 2 = does not live alone) | | | Education | | |
| IVs | B  (*SE*) | *p* | 95% CI for B | B  (*SE*) | *p* | 95% CI for B | B  (*SE*) | *p* | 95% CI for B | B  (*SE*) | *p* | 95% CI for B |
| Life satisfaction | 0.13 (0.04) | <.002 | 0.05, 0.21 | -0.54 (0.57) | .346 | -1.67, 0.59 | 0.86 (0.57) | .123 | -0.25, 1.97 | -0.26 (0.26) | .318 | -0.78, 0.26 |
| Self-esteem | 0.01 (0.07) | .355 | -0.01, -0.02 | 0.06 (0.10) | .525 | -0.13, 0.23 | 0.09 (0.04) | .032 | 0.01, 0.17 | 0.03 (0.04) | .434 | -0.05, 0.12 |
| Purpose in life | 0.01 (0.00) | .012 | 0.00, 0.01 | -0.06 (0.04) | .135 | -0.14, 0.02 | 0.14 (0.09) | .139 | -0.04, 0.32 | 0.01 (0.02) | .690 | -0.03, 0.04 |
| Social support | 0.01 (0.00) | .007 | 0.00, 0.02 | -0.11 (0.05) | .030 | -0.20, -0.01 | 0.03 (0.05) | .531 | -0.07, 0.13 | -0.00 (0.02) | .946 | -0.05, 0.04 |
| Self-rated health | 0.14 (0.06) | .015 | 0.03, 0.25 | 0.57 (0.72) | .425 | -0.84, 1.98 | -0.63 (0.71) | .373 | -2.03, 0.76 | -0.15 (0.33) | .651 | -0.79, 0.50 |
| Self-efficacy | 0.02 (0.01) | .030 | 0.00, 0.04 | -0.12 (0.13) | .339 | -0.37, 0.13 | 0.11 (0.12) | .359 | -0.13, 0.35 | -0.04 (0.05) | .433 | -0.15, 0.06 |
| Physical activity | 0.03 (0.02) | .217 | -0.12, 0.08 | -0.03 (0.32) | .917 | -0.66, 0.60 | 0.42 (0.33) | .202 | -0.23, 1.07 | 0.09 (0.15) | .580 | -0.22, 0.39 |
| Personal growth | -0.01 (0.00) | .014 | 0.00, 0.02 | -0.04 (0.05) | .417 | -0.15, 0.06 | 0.06 (0.06) | .283 | -0.05, 0.17 | -0.04 (0.03) | .118 | -0.09, 0.01 |
| Living arrangement | 0.00 (0.09) | .975 | -0.18, 0.18 | 1.83 (1.35) | .176 | -0.82, 4.48 | - | - | - | 0.36 (0.56) | .522 | -0.75, 1.47 |
| Education | 0.00 (0.04) | .914 | -0.08, 0.09 | -0.06 (0.57) | .923 | -1.17, 1.06 | 0.36 (0.56) | .522 | -0.75, 1.47 | - | - | - |

*Note*. IVs = independent variables; B = unstandardized estimates; SE = standard error of B; *p* = significance value; CI = confidence interval. Bonferroni-adjusted alpha level of < .005.


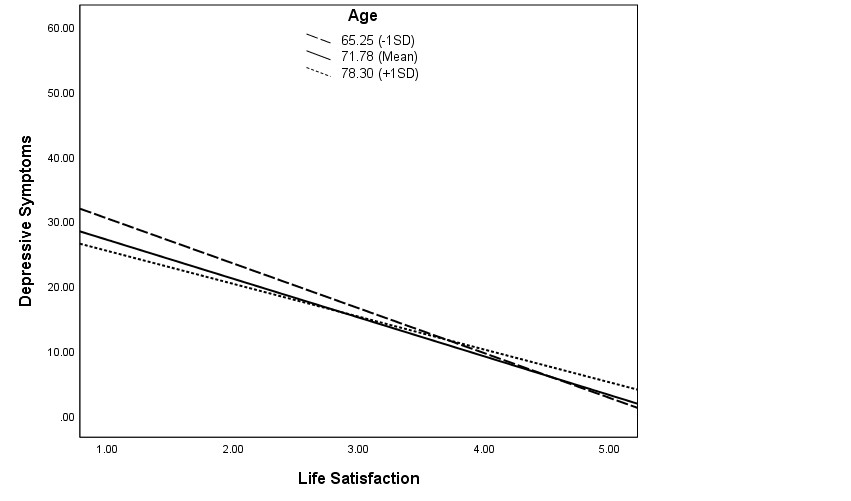


Figure S1. *Significant moderating effect of age on the relationship between life satisfaction and depressive symptoms.*
